# BMAL1 regulates *Propionibacterium acnes*-induced skin inflammation via REV-ERBα in mice

**DOI:** 10.7150/ijbs.71719

**Published:** 2022-03-21

**Authors:** Feng Li, Luomin Lin, Yiting He, Guanghui Sun, Dong Dong, Baojian Wu

**Affiliations:** 1 College of pharmacy, Jinan University, Guangzhou 510632, China.; 2 School of Medicine, Jinan University, Guangzhou, China.; 3 Institute of Molecular Rhythm and Metabolism, Guangzhou University of Chinese Medicine, Guangzhou, China.

**Keywords:** Acne vulgaris, BMAL1, REV-ERBα, skin inflammation, circadian rhythms, *P. acnes*

## Abstract

Acne vulgaris is a common skin disease, affecting over 80% of adolescents. Inflammation is known to play a central role in acne development. Here, we aimed to investigate the role of the central clock gene *Bmal1* in acne-associated inflammation in mice. To this end, mice were injected intradermally with *Propionibacterium acnes* (*P. acnes*) to induce acne-associated skin inflammation. We found that *Bmal1* and its target genes *Rev-erbα, Dbp, Per1* and* Cry2* were down-regulated in the skin of *P. acnes*-treated mice, suggesting a role of *Bmal1* in the condition of acne. Supporting this, *Bmal1*-deleted or jet-lagged mice showed exacerbated *P. acnes*-induced inflammation in the skin. Regulation of *P. acnes*-induced inflammation by *Bmal1* was further confirmed in RAW264.7 cells and primary mouse keratinocytes. Transcriptomic and protein expression analyses suggested that *Bmal1* regulated *P. acnes*-induced inflammation via the NF-κB/NLRP3 axis, which is known to be repressed by REV-ERBα (a direct target of BMAL1). Moreover, loss of *Rev-erbα* in mice exacerbated *P. acnes*-induced inflammation. In addition, *Rev-erbα* silencing attenuated the inhibitory effects of *Bmal1* on *P. acnes*-induced inflammation. *Bmal1* knockdown failed to modulate *P. acnes*-induced inflammation in *Rev-erbα*-silenced cells. It was thus proposed that *Bmal1* restrained *P. acnes*-induced skin inflammation via its target REV-ERBα, which acts on the NF-κB/NLRP3 axis to repress inflammation. In conclusion, *Bmal1* disruption is identified as a potential pathological factor of acne-associated inflammation. The findings increase our understanding of the crosstalk between skin clock and acne and suggest targeting circadian rhythms as a promising approach for management of acne.

## Introduction

Acne vulgaris is a common skin disease associated with obstruction and inflammation of pilosebaceous units, affecting over 80% of adolescents and about two-thirds of adults 1. Acne presents as the lesions such as comedones, papules, pustules and cysts, and typically develops on the face, neck and chest 2. Although acne may be commonly regarded as a cosmetic concern, the disease can result in permanent scarring, and even disfigurement, and is also correlated with various psychological problems such as depression, anxiety, frustration, and social isolation 3. The pathogenesis of acne vulgaris is incompletely understood, however, several contributing factors have been described, including excess sebum production, follicular hyperkeratinization, formation of *Propionibacterium* colonies, and inflammation 4. Of note, inflammation plays a central role as it occurs in early and late stages of acne vulgaris 5. Treatment of acne involves a variety of topical and systemic medications such as antibiotics. However, many patients are contraindicated against or refractory to these medications 6. Therefore, it is of interest to understand the molecular events underlying acne development and to search for novel therapeutic strategies.

All living organisms are subject to circadian rhythms which are orchestrated by circadian clocks. In mammals, the clock system is hierarchical and constituted by the central pacemaker (also known as master clock) located in the suprachiasmatic nucleus (SCN) of the hypothalamus and peripheral clocks present in peripheral tissues such as liver, kidney, lung and heart 7. The SCN master clock receives light input signal from the retina and is synchronized to the external solar time 8. The master clock further coordinates peripheral clocks through both neural and hormonal pathways 9. At the molecular level, mammal circadian clock consists of several genes and their proteins that function together to generate robust 24-h rhythmicity in gene expression using a negative feedback mechanism 8. Of note, the core components BMAL1 (brain and muscle ARNT-like protein 1) and CLOCK (circadian locomotor output cycles kaput) form a heterodimer to activate the transcription of clock-controlled genes (CCGs) including *Pers* (periods) and *Crys* (cryptochromes) that contain E-box elements in their promoter and/or enhancer regions 8,10 . At a later stage of the cycle, PER and CRY proteins in turn repress the activity of CLOCK-BMAL1 by forming a complex, leading to down-regulation of *Pers* and *Crys* as well as other CCGs 11. Once PER and CRY proteins are degraded, a new cycle of CLOCK-BMAL1-mediated transcription can begin 12.

Many aspects of the skin display circadian variations, including transepidermal water loss, skin blood flow, skin permeability, and keratinocyte proliferation, suggesting a tight association of the skin function with circadian clock 13. It is thus of no surprise that several types of skin diseases have been linked to disruption of circadian rhythms caused by either genetic or environmental factors. For instance, *Per2* mutant mice are prone to imiquimod-induced dermatitis 14. Loss of *Clock* gene in mice leads to more severe delayed-type skin allergic reactions 15. Night-shift workers show an increased incidence of psoriasis 16. Interestingly, circadian dyssynchrony may also affect the development of acne as suggested by a significant difference on acne between the rotating shift work nurses and the nurses on a fixed day schedule 17. However, the underlying mechanisms for clock regulation of acne and associated conditions remain unknown.

In the present study, we aimed to investigate the role of the central clock gene *Bmal1* in acne-associated inflammation in mice. To this end, mice were injected intradermally with *P. acnes* to induce acne-associated skin inflammation. We found that *Bmal1* and its target genes *Rev-erbα, Dbp, Per1* and* Cry2* were down-regulated in the skin of *P. acnes*-treated mice, suggesting a role of *Bmal1* in the condition of acne. Supporting this, *Bmal1*-deleted or jet-lagged mice showed exacerbated *P. acnes*-induced inflammation in the skin. Regulation of *P. acnes*-induced inflammation by *Bmal1* was further confirmed in RAW264.7 cells and primary mouse keratinocytes. Mechanistically, *Bmal1* regulated *P. acnes*-induced skin inflammation via its target REV-ERBα, which acts on the NF-κB/NLRP3 axis to repress inflammation. Overall, our study provides a molecular mechanism for regulation of acne-associated inflammation by the skin clock, and highlights disruption of circadian rhythms as a pathogenetic factor to acne development.

## Materials and Methods

Anti-BMAL1 (14268-1-AP), anti-REV-ERBα (14506-1-AP), anti-p65 (10745-1-AP) and anti-β-actin (20536-1-AP) antibodies were purchased from Proteintech (Wuhan, China). Anti-NLRP3 (15101), anti-ASC (67824) and anti-p-p65 (3031) antibodies were obtained from Cell Signaling Technology (Beverly, MA). Anti-pro-caspase-1 (14F468) antibody was obtained from Santa Cruz Biotechnology (Santa Cruz, CA). Anti-IL-1β (AF-401) antibody was purchased from R&D systems (Minneapolis, MN). Anti-PAB antibody (D371-3) was purchased from Medical & Biological Laboratories (Nagoya, Japan). pcDNA3.1, pcDNA3.1-*Bmal1*, pcDNA3.1-*Rev-erbα*, siBmal1 (siRNA targeting *Bmal1*), siRev-erbα (siRNA targeting *Rev-erbα*) and a negative control for siRNAs (siNC) were obtained from Transheep Technologies (Shanghai, China).

### Mice

Wild-type (WT) C57BL/6 mice were obtained from HFK Bioscience (Beijing, China). *Bmal1*^-/-^ and *Rev-erbα*^-/-^ mice (C57BL/6 background) have been established and validated in our laboratory 18,19. Primers used for genotyping are listed in Table 1. All mice were maintained under a 12 h light/12 h dark cycle (light on at 7:00 AM and light off at 7:00 PM), with free access to food and water. Animal experimental procedures were approved by Institutional Animal Care and Use Committee of Guangzhou University of Chinese Medicine and were performed in accordance with the NIH Guide for the Care and Use of Laboratory Animals.

### Propionibacterium acnes (P. acnes)

*P. acnes* was obtained from Guangdong Microbial Culture Collection Center (Guangzhou, China), and cultured in Gifu Anaerobic Medium under an anaerobic condition with AnaeroPack-Anaero (Mitsubishi Gas Chemical, Tokyo, Japan) at 37°C for 24 h. The culture was centrifuged at 5,000 g for 15 min. The pellets (*P. acnes*) were collected and heat-inactivated at 95°C for 5 min.

### *P. acnes*-induced skin inflammation

Mice with *P. acnes*-induced skin inflammation were established as previously described 20. In brief, mice were shaved at the back with an area of about 4 cm^2^, and injected intradermally with approximately 2×10^10^ CFU *P. acnes* in 20 µl phosphate-buffered saline (PBS). Control mice were injected intradermally with vehicle. Two weeks later, skin samples were obtained by excisional biopsy of the inflammatory nodule. Mice were kept under constant darkness one day prior to sample collection.

### H&E staining

Skin tissues were fixed in 4% paraformaldehyde for 24 h, paraffin-embedded, and cut into 5 µm sections. The sections were stained with hematoxylin and eosin (H&E), and then visualized by a Nikon Eclipse Ci-L microscope (Tokyo, Japan). Inflammation was scored as follows: grade 0, no changes; grade 1, infiltration of a few cells; grade 2, moderate infiltration; and grade 3, extensive infiltration.

### Immunohistochemistry (IHC)

Skin tissues were fixed in 4% paraformaldehyde and embedded in paraffin. 5 µm paraffin-embedded sections were heated at 65°C for 1 hour, dewaxed in xylene, and rehydrated in descending concentrations of ethanol. Heat-induced antigen retrieval was performed by boiling samples at 100°C in 0.01 M citrate buffer solution (pH 6.0) for 10 min. The sections were pre-blocked with 5% goat serum and incubated with anti-PAB antibody overnight at 4°C. After washing with PBS, the sections were incubated with an anti-mouse horseradish peroxidase antibody at room temperature for 0.5 h at 37°C, followed by staining with diaminobenzidine tetrahydrochloride and counterstaining with hematoxylin. The sections were imaged with a Nikon Eclipse Ti-SR microscope (Tokyo, Japan). IHC staining intensities were divided into four grades as follows: 0 (negative), 1 (weak), 2 (moderate), and 3 (strong). The average staining percentage was scored as follows: 1 (< 25%), 2 (25-50%), 3 (51-75%), and 4 (> 75%). The IHC scores were then determined by multiplying the intensity score by the staining percentage score.

### Isolation of primary mouse keratinocytes

Primary mouse keratinocytes were isolated from newborn mice as previously described 21. The newborn mice were sacrificed by rapid cervical dislocation. Trunk skin was peeled off as a single piece, and incubated with 4 mg/ml dispase overnight at 4°C. On the next day, the epidermis was separated from the dermis using forceps, and incubated with 0.25% trypsin. After 20 min, the epidermis was rubbed with forceps to release the cells, and filtered through a 100 μm strainer. Cell suspension was centrifuged for 5 min at 180 g, and keratinocytes were collected. The cells were then seeded into collagen-coated plates, and cultured in keratinocyte growth medium.

### Cell transfection and treatment

RAW264.7 cells were obtained from National Collection of Authenticated Cell Cultures (Shanghai, China), and cultured in Dulbecco's modified Eagle's medium (DMEM; Gibco, Grand Island, NY) with 10% fetal bovine serum (Gibco, Grand Island, NY). RAW264.7 cells and primary mouse keratinocytes were transfected with pcDNA3.1-*Bmal1*, pcDNA3.1-*Rev-erbα*, siBmal1, siRev-erbα or control using the jetPRIME transfection reagent (Polyplus Transfection, Illkirch, France). On next day, cells were treated with heated-killed *P. acnes*. After 6 h, cells were collected for qRT-PCR or Western blotting.

### RNA-sequencing (RNA-seq)

Skin tissues collected from WT mice, *P. acnes*-treated WT mice and *P. acnes*-treated *Bmal1*^-/-^ mice were used for RNA-seq. RNA was extracted using RNAiso Plus reagent and analyzed for quality using Agilent 2100 BioAnalyzer Expert (Santa Clara, CA). Library preparation was performed using NEBNext Ultra II Directional RNA Library Prep Kit for Illumina (Ipswich, MA). The cDNA libraries were sequenced on Illumina HiSeq X Ten to generate 2 × 150-base pair (bp) paired-end reads. Reads were aligned to the mm10 reference genome (UCSC) using STAR. Differentially expressed genes were obtained using DESeq2 (a RNA-seq data analysis software package). Genes with adjusted P ≤ 0.05 were considered as differentially expressed. Heat maps were plotted using the pheatmap package version 1.0.10.

### RT-qPCR (reverse transcription-quantitative polymerase chain reaction)

Total RNA was isolated using the Trizol reagent (Accurate Biotechnology, Hunan, China). Total RNA was reversely transcribed into cDNA by using a PrimeScript RT Master Mix reagent kit (Takara Biotech, Osaka, Japan). qPCR reactions were performed using SYBR Premix Ex Taq (Takara Biotech, Osaka, Japan) with a TProfessional Thermocycler (Biometra, Goettingen, Germany). The amplification program consisted of one cycle at 95°C for 5 min, 40 cycles at 94°C for 15 s, annealing at 60°C for 30 s, and extension at 72°C for 30 s. Primer sequences are listed in Table 1.

### Western blotting

Protein concentration was measured using a BCA reagent kit (Beyotime, Shanghai, China). Protein samples were subjected to sodium dodecyl sulphate-polyacrylamide gel electrophoresis (10% gel), and transferred onto polyvinylidene fluoride membrane. After blocking with 5% skim milk, the membrane was incubated with a primary antibody (anti-p65, anti-p-p65, anti-NLRP3, anti-pro-caspase-1, anti-ASC or anti-IL-1β) overnight at 4°C, followed by incubation with a horseradish peroxidase-conjugated secondary antibody. Protein bands were imaged by using the Omega Lum G imaging system (Aplegen, Pleasanton, CA).

### Statistical analysis

Data are presented as mean ± SD (standard deviation). Student's t-test was used to analyze a statistical difference between two groups. Two-way ANOVA followed by Bonferroni post hoc test was used for multiple group comparisons. The level of significance was set at p < 0.05 (*).

## Results

### *Bmal1* is dysregulated in the skin of mice treated with *P. acnes*

**Figure 1:**
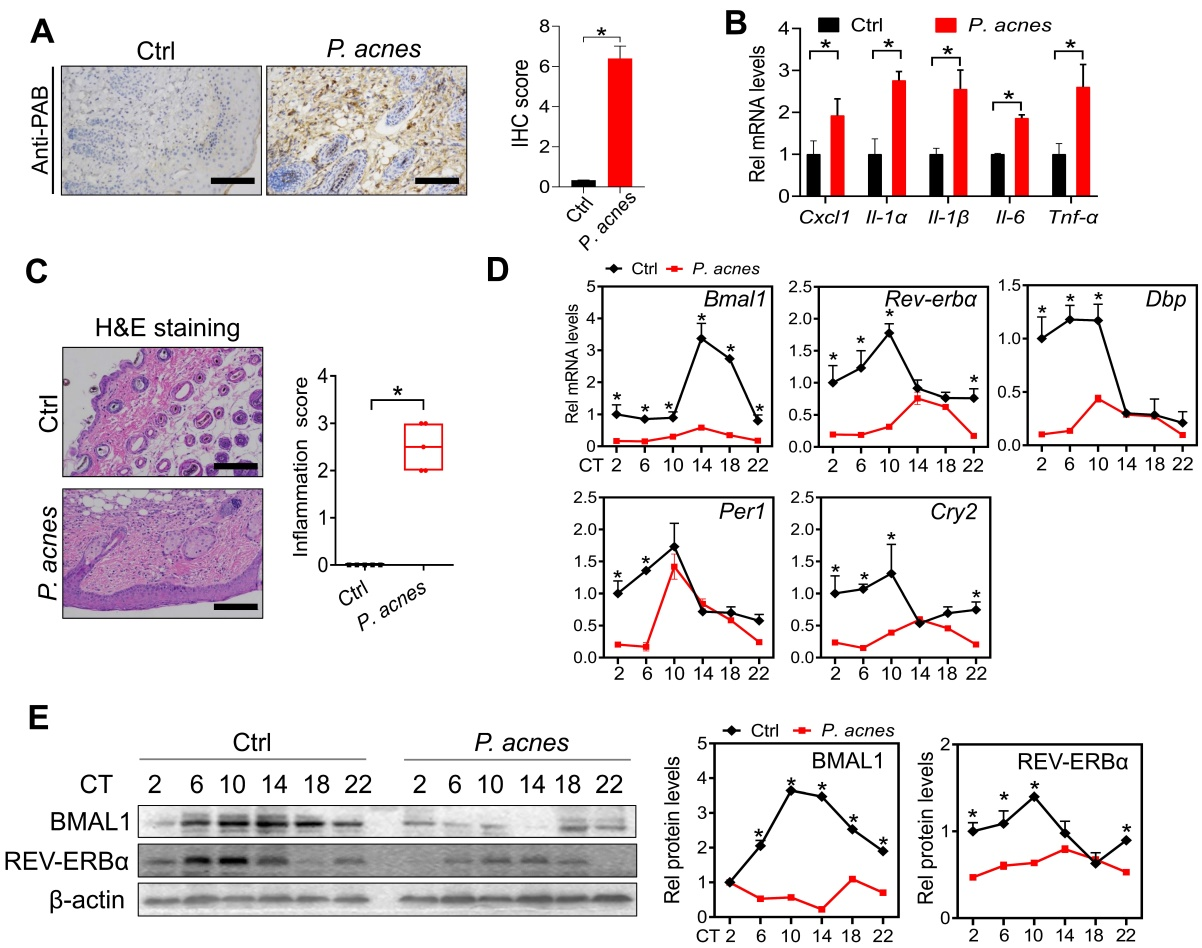
** Clock genes are dysregulated in the skin of mice treated with *P. acnes*. (A)** IHC staining of the skin of *P. acnes-*injected and control mice against PAB and IHC score*.* Scale bar = 100 μm. Data are mean ± SD (*n* = 5). *p < 0.05 (t-test).** (B)** mRNA expression of* Cxcl1*, *Il-1α*, *Il-1β*, *Il-6* and *Tnf-α* in the skin derived from *P. acnes-*injected and control mice*.* Data are mean ± SD (*n* = 5). *p < 0.05 (t-test).** (C)** H&E staining (left panel) and inflammation score (right panel) of skin derived from *P. acnes*-injected and control mice*.* Scale bar = 100 μm. Data are mean ± SD (*n* = 5). *p < 0.05 (t-test). **(D)** mRNA expression of* Bmal1*, *Rev-erbα*, *Dbp*, *Per1* and *Cry2* in the skin derived from *P. acnes*-injected and control mice at 6 circadian time points (CT2, CT6, CT10, CT14, CT18 and CT22). Data are mean ± SD (*n* = 5). *p < 0.05 as determined by two-way ANOVA and Bonferroni post hoc test. **(E)** Protein bands (left panel) and quantitative data (right panel) of BMAL1 and REV-ERBα in the skin derived from *P. acnes*-injected and control mice at 6 circadian time points. Data are mean ± SD (*n* = 5). *p < 0.05 as determined by two-way ANOVA and Bonferroni post hoc test. Ctrl, control; Rel, relative; CT, circadian time.

Mice were treated with *P. acnes* via intradermal injection to induce skin inflammation. Immunohistochemical staining with PAB antibody showed a number of *P. acnes*-positive cells in the skin of treated mice (Fig. 1A). As expected, *P. acnes* treatment activated the inflammatory responses in the skin, as evidenced by elevated levels of the pro-inflammatory factors including *Cxcl1*, *Il-1α*, *Il-1β*,* Il-6* and *Tnf-α* (Fig. 1B). This was supported by H&E staining which revealed significant inflammation in the skin (Fig. 1C). To examine whether core clock genes are affected by *P. acnes* treatment, skin samples were collected at six different circadian time points with an interval of 4 hours. We found that *Bmal1* and its target genes *Rev-erbα, Dbp, Per1* and* Cry2* were down-regulated at the mRNA level in the skin (Fig. 1D). In line with this, BMAL1 and REV-ERBα proteins were reduced in the skin of *P. acnes*-treated mice (Fig. 1E). Because REV-ERBα is a known inflammatory modulator, our findings suggest a potential role of BMAL1 in *P. acnes*-induced skin inflammation.

### Loss of *Bmal1* in mice exacerbates *P. acnes*-induced skin inflammation

**Figure 2:**
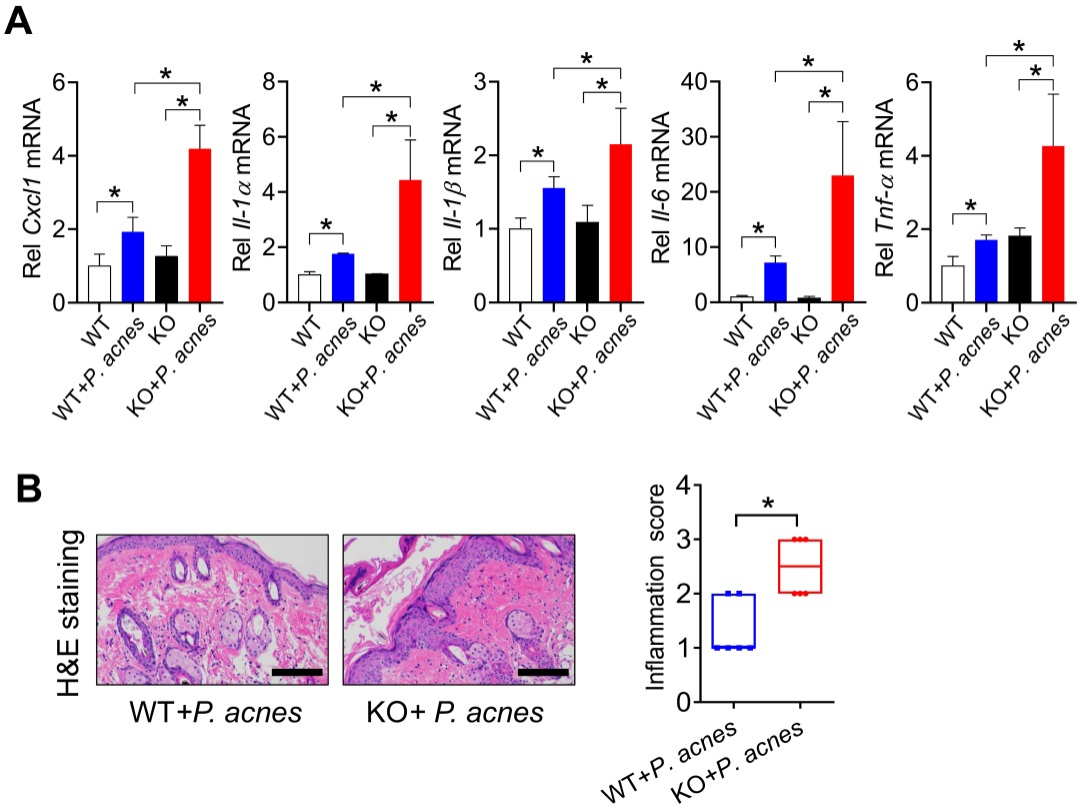
** Loss of *Bmal1* in mice exacerbates *P. acnes*-induced skin inflammation. (A)** mRNA expression of* Cxcl1*, *Il-1α*,* Il-1β*, *Il-6* and *Tnf-α* in the skin derived from* P. acnes*-injected WT (wild-type) and KO (*Bmal1* knockout) mice. Data are mean ± SD (*n* = 6). *p < 0.05 as determined by two-way ANOVA and Bonferroni post hoc test. **(B)** H&E staining (left panel) and inflammation score (right panel) of skin derived from *P. acnes*-injected WT and KO mice*.* Scale bar = 100 μm. Data are mean ± SD (*n* = 6). *p < 0.05 (t-test).

We next investigated the role of BMAL1 in *P. acnes*-induced skin inflammation using *Bmal1* knockout (KO) mice. Both WT and KO mice were treated with *P.acnes* to induce skin inflammation. Compared with WT mice, KO mice showed an increased susceptibility to *P. acnes*-induced skin inflammation, as evidenced by higher levels of pro-inflammatory factors such as *Cxcl1*, *Il-1α*, *Il-1β*,* Il-6* and *Tnf-α* in the knockout mice (Fig. 2A). H&E staining also revealed more extensive inflammation in the skin of KO mice (Fig. 2B). These findings indicated that *Bmal1* has a critical role in *P.acnes-*induced skin inflammation.

### Jet-lagged mice show exacerbated *P. acnes*-induced skin inflammation

**Figure 3:**
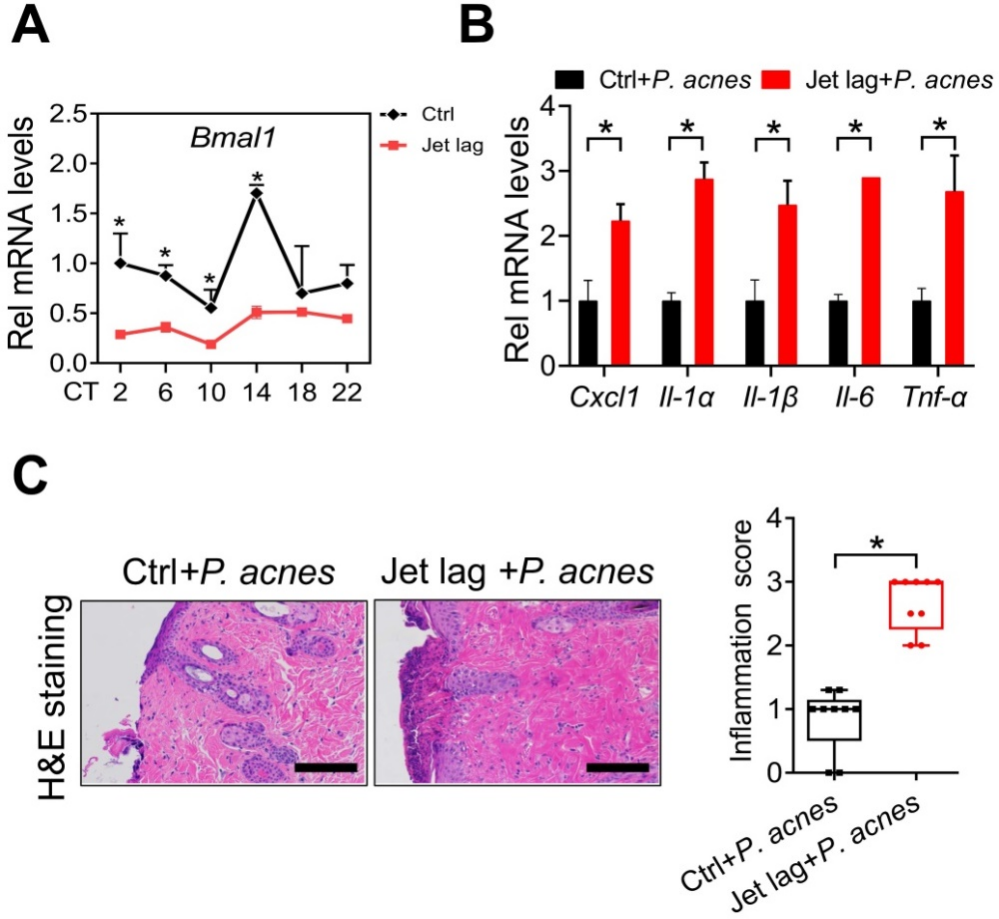
** Jet-lagged mice show exacerbated *P. acnes*-induced skin inflammation. (A)** mRNA expression of *Bmal1* in the skin derived from jet-lagged and control mice at 6 circadian time points (CT2, CT6, CT10, CT14, CT18 and CT22). *p < 0.05 as determined by two-way ANOVA and Bonferroni post hoc test. **(B)** mRNA expression of* Cxcl1*, *Il-1α*, *Il-1β*,* Il-6* and* Tnf-α* in the skin derived from Jet-lagged and control mice treated with *P. acnes*. *p < 0.05 (t-test). **(C)** H&E staining (left panel) and inflammation score (right panel) of skin derived from Jet-lagged and control mice treated with *P. acnes*. Scale bar = 100 μm. *p < 0.05 (t-test). Data are mean ± SD (*n* = 9).

Jet lag is a model of physiological disturbance in circadian rhythms 22,23. Jet-lagged humans and animals show disrupted expression of clock genes 24,25. We thus examined whether jet lag has an effect on the development of skin inflammation. To this end, mice were subjected to a jet lag schedule of 8 h light advance every 2 days for 10 days as previously described 26. We confirmed that *Bmal1* was disrupted in the skin of jet-lagged mice (Fig. 3A). Both jet-lagged and normal mice were then treated with *P.acnes* to induce skin inflammation. Compared with normal mice, jet-lagged mice were more sensitive to *P. acnes*-induced skin inflammation, as evidenced by higher levels of pro-inflammatory factors such as *Cxcl1*, *Il-1α*, *Il-1β*,* Il-6* and *Tnf-α* (Fig. 3B). H&E staining confirmed more extensive inflammation in the skin of jet-lagged mice (Fig. 3C). These data support a regulatory role of the central clock gene *Bmal1* in *P.acnes-*induced skin inflammation.

### *Bmal1* regulates *P. acnes*-induced inflammation in RAW264.7 cells and primary mouse keratinocytes

**Figure 4:**
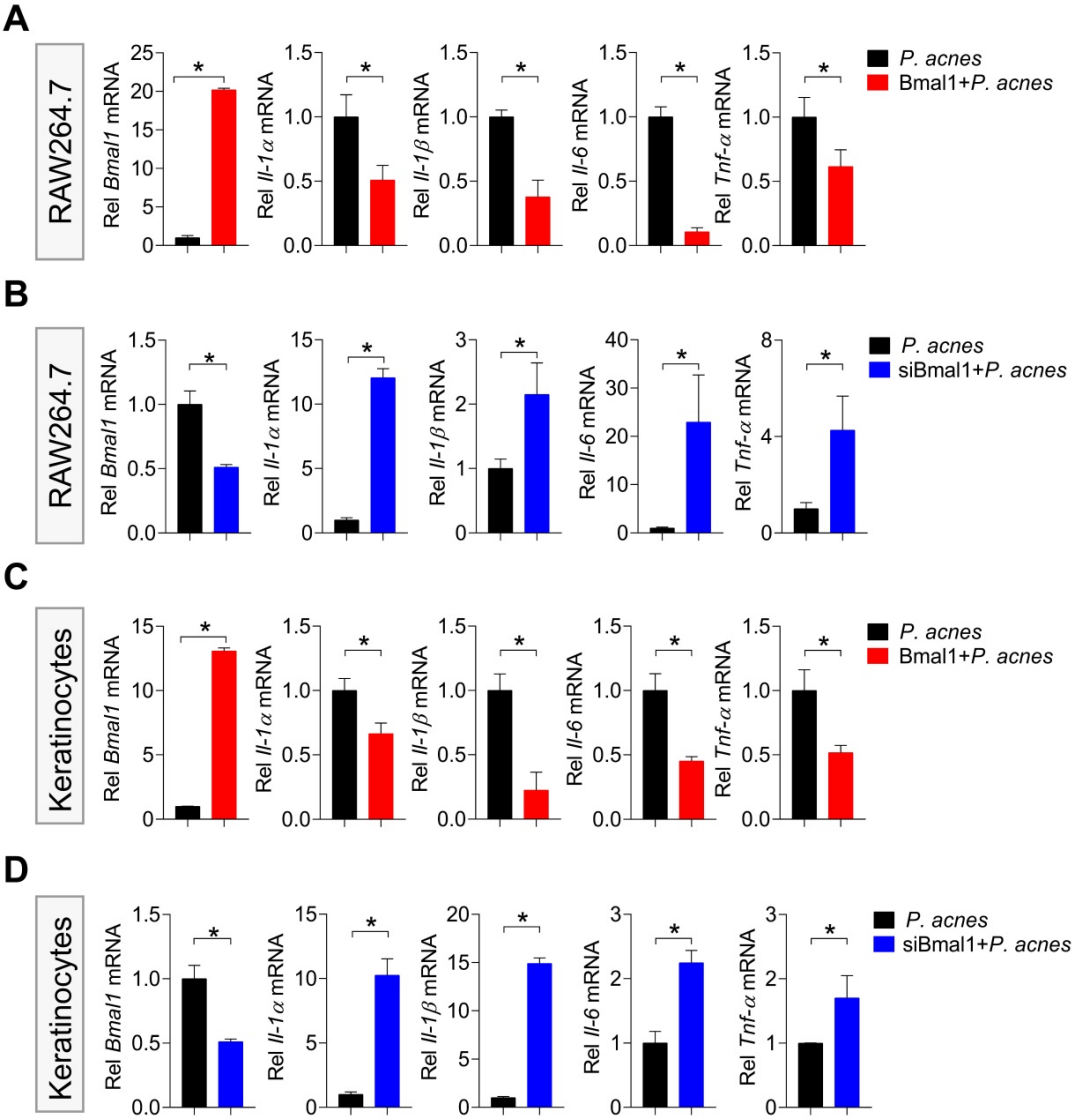
**Bmal1 regulates P. acnes-induced inflammation in RAW264.7 cells and primary mouse keratinocytes. (A)** Effects of Bmal1 overexpression on Bmal1, Il-1α, Il-1β, Il-6 and Tnf-α mRNA expression in RAW264.7 cells after treatment with P. acnes for 6 h. Data are mean ± SD (n = 3). *p < 0.05 (t-test). **(B)** Effects of Bmal1 knockdown on Bmal1, Il-1α, Il-1β, Il-6 and Tnf-α mRNA expression in RAW264.7 cells after treatment with P. acnes for 6 h. Data are mean ± SD (n = 3). *p < 0.05 (t-test). **(C)** Effects of Bmal1 overexpression on Bmal1, Il-1α, Il-1β, Il-6 and Tnf-α mRNA expression in primary mouse keratinocytes after treatment with P. acnes for 6 h. Data are mean ± SD (n = 3). *p < 0.05 (t-test). **(D)** Effects of Bmal1 knockdown on Bmal1, Il-1α, Il-1β, Il-6 and Tnf-α mRNA expression in primary mouse keratinocytes after treatment with P. acnes for 6 h. Data are mean ± SD (n = 3). *p < 0.05 (t-test).

Next, we examined the effects of *Bmal1* on* P. acnes*-induced inflammation in RAW264.7 cells and primary mouse keratinocytes. *Bmal1*-overexpressed RAW264.7 cells (using the overexpression plasmid of *Bmal1*) showed decreased expression of the inflammatory cytokines such as *Il-1α*, *Il-1β*, *Il-6* and *Tnf-α* compared to control cells (Fig. 4A). Consistently, *Bmal1*-silenced RAW264.7 cells (using a specific siRNA) had increased expression of *Il-1α*, *Il-1β*, *Il-6* and *Tnf-α* (Fig. 4B). Similar effects of *Bmal1* on pro-inflammatory genes were observed in primary mouse keratinocytes (Fig. 4C/D). These findings support a role of *Bmal1* in regulation of *P. acnes*-induced inflammation.

### *Bmal1* regulates skin inflammation via the NF-κB/NLRP3 axis

**Figure 5:**
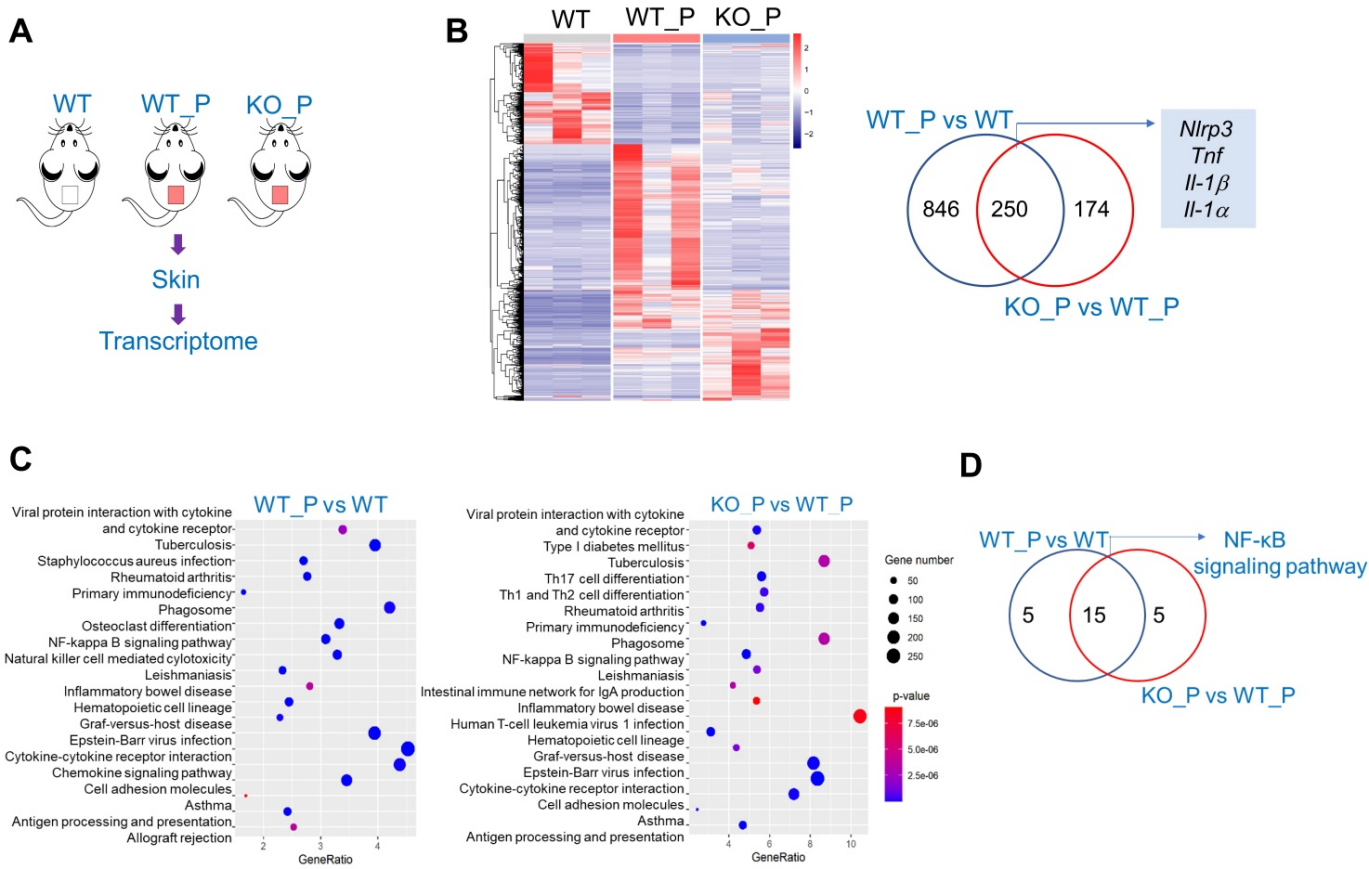
**RNA-sequencing analyses for deferentially expressed genes (DEGs) in WT, WT_P and KO_P mice. (A)** Schematic diagram showing the protocol for RNA-sequencing. **(B)** Heat map for mRNA expression profiles (left panel) and Venn diagram (right panel) for DEGs in the skin derived from WT, WT_P and KO_P mice. **(C)** KEGG pathway analysis of DEGs in the skin derived from WT, WT_P and KO_P mice. **(D)** Venn diagram for KEGG pathways associated with DEGs in the skin derived from WT, WT_P and KO_P mice. WT, wild-type mice. WT_P, wild-type mice treated with* P. acnes*. KO_P,* Bmal1^-/-^* mice treated with* P. acnes*.

To explore the mechanism by which *Bmal1* regulates *P. acnes*-induced inflammation, we performed transcriptomic analyses using skin samples from WT (wild-type), WT_P (wild-type treated with *P. acnes*) and KO_P (*Bmal1^-/ -^* mice treated with* P. acnes*) mice (Fig. 5A). 1096 DEGs (deferentially expressed genes) were identified when WT_P samples were compared to WT samples, and 424 DEGs were identified when KO_P samples were compared to WT_P samples (Fig. 5B). Of the DEGs associated with both *P. acnes* and *Bmal1* knockout, we identified several genes involved in NF-κB and NLRP3 inflammasome pathways, including *Nlrp3*, *Tnf*, *Il-1β* and *Il-1α* (Fig. 5B). KEGG pathway analyses revealed that *P. acnes*-induced and *Bmal1*-regulated inflammations were associated with activation of NF-κB and NF-κB-related signaling pathways (such as tuberculosis, rheumatoid arthritis, leishmaniasis, inflammatory bowel disease, epstein-barr virus infection, and asthma) (Fig. 5C). We also observed a high overlap (75%) between *P. acnes-* and *Bmal1-*associated enriched pathways, supporting regulation of *P. acnes*-induced skin inflammation by *Bmal1* (Fig. 5D).

**Figure 6:**
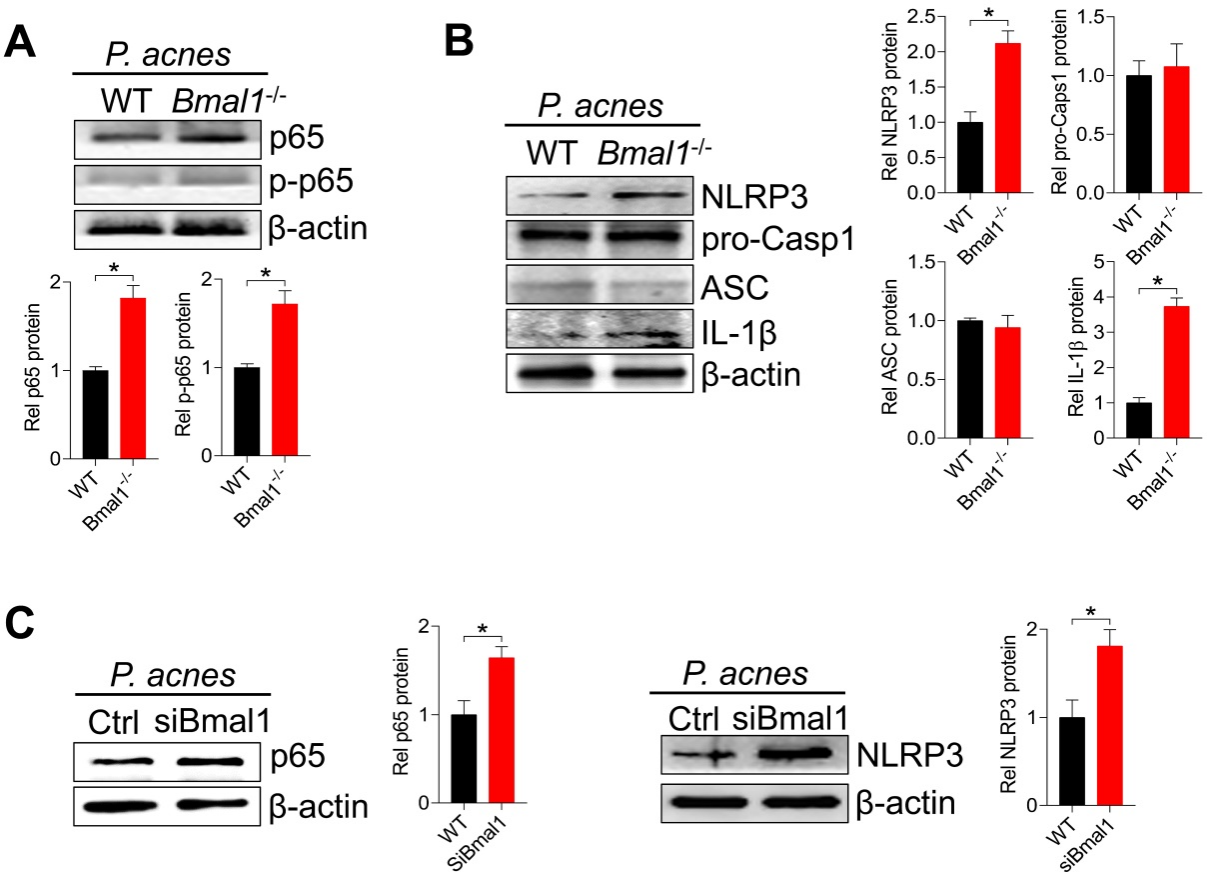
***Bmal1* regulates skin inflammation via the NF-κB/NLRP3 axis**. **(A)** Protein bands (top panel) and quantitative data (bottom panel) of p65 and p-p65 in the skin derived from *P. acnes*-injected WT and* Bmal1^-/-^* mice. Data are mean ± SD (*n* = 6). *p < 0.05 (t-test). **(B)** Protein bands (left panel) and quantitative data (right panel) of NLRP3, pro-Casp1, ASC and IL-1β in the skin from *P. acnes*-injected WT and *Bmal1^-/-^* mice. Data are mean ± SD (*n* = 6). *p < 0.05 (t-test). **(C)** Effects of *Bmal1* knockdown on p65 protein (left panel) and NLRP3 protein (right panel) in primary mouse keratinocytes after treatment with *P. acnes* for 6 h. Data are mean ± SD (*n* = 3). *p < 0.05 (t-test). pro-Casp1, pro-caspase1.

We further examined the effects of *Bmal1* on the protein expression of NF-κB (p65) and NLRP3 inflammasome components (i.e., NLRP3, ASC, and pro-Casp1) in mice treated with *P. acnes*. *Bmal1* ablation led to elevated levels of total p65 protein and phosphorylated p65 (p-p65) in the skin of mice (Fig. 6A). In the meantime, NLRP3 protein was higher in KO than in WT mice, but ASC and pro-Casp1 proteins remained unchanged (Fig. 6B). We also found an elevated protein level of IL-1β, an indicator of NLRP3 inflammasome activation (Fig. 6B). In addition, *Bmal1* knockdown caused significant increases in p65 and NLRP3 proteins in the primary mouse keratinocytes stimulated with *P. acnes* (Fig. 6C). Taken together, *Bmal1* regulates *P. acnes*-induced skin inflammation via the NF-κB/NLRP3 axis.

### Loss of *Rev-erbα* in mice exacerbates *P. acnes*-induced skin inflammation

**Figure 7:**
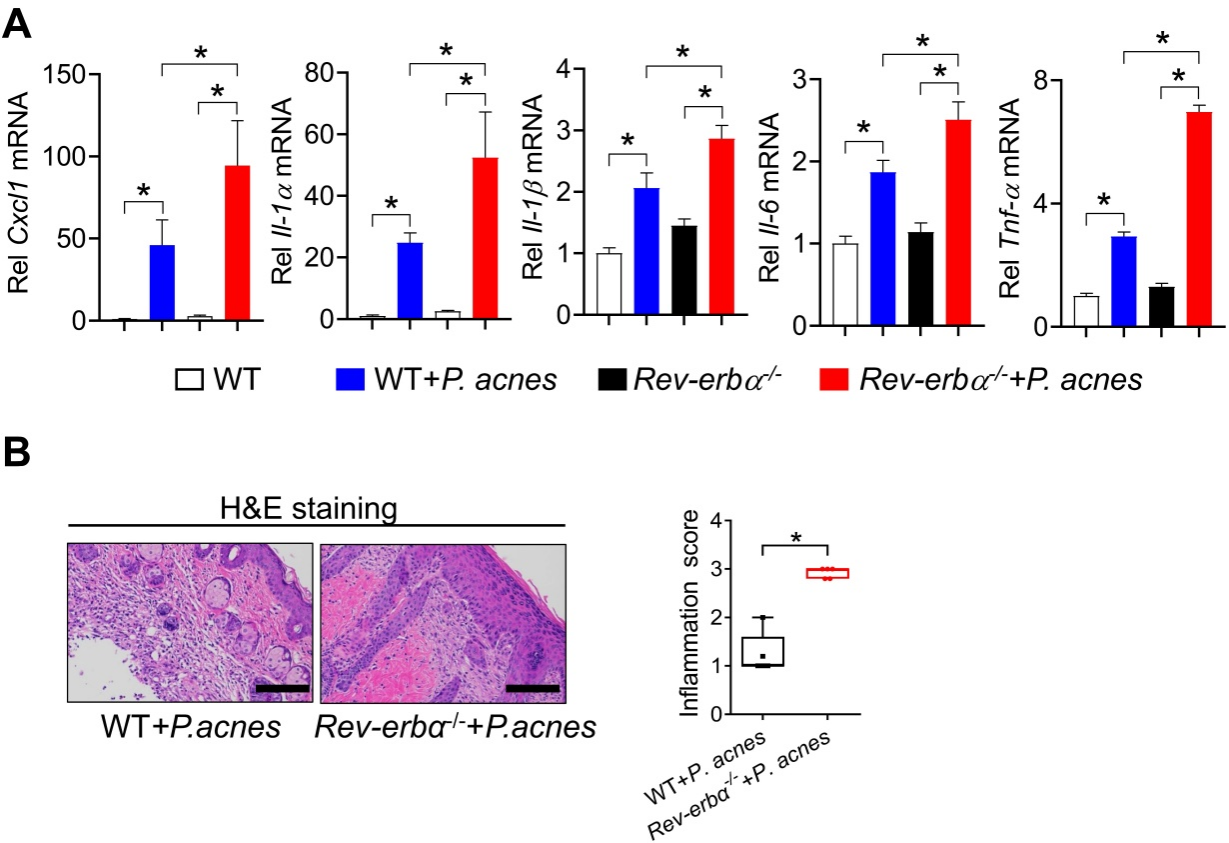
** Loss of *Rev-erbα* in mice exacerbates *P. acnes*-induced skin inflammation. (A)** mRNA expression of *Cxcl1*, *Il-1α*, *Il-1β*, *Il-6* and *Tnf-α* in the skin derived from *P. acnes*-injected *Rev-erbα*^-/-^ and WT mice. Data are mean ± SD (*n* = 5). *p < 0.05 as determined by two-way ANOVA and Bonferroni post hoc test. **(B)** H&E staining (left panel) and inflammation score (right panel) of the skin derived from *P. acnes*-injected *Rev-erbα*^-/-^ and WT mice. Data are mean ± SD (*n* = 5). *p < 0.05 (t-test).

We have previously shown that REV-ERBα, a direct target of BMAL1, represses colonic inflammation through the NF-κB/NLRP3 axis using a transrepression mechanism 19. Given that the NF-κB/NLRP3 axis is also involved in BMAL1 regulation of *P. acnes*-induced skin inflammation, we hypothesized that REV-ERBα may mediate the regulation of skin inflammation by BMAL1. We thus investigated whether REV-ERBα does have a role in *P. acnes*-induced skin inflammation. Compared with wild-type mice, *Rev-erbα^-/-^* mice showed an increased sensitivity to *P. acnes*-induced skin inflammation, as evidenced by higher levels of pro-inflammatory factors such as *Cxcl1*, *Il-1α*, *Il-1β*,* Il-6* and *Tnf-α* in the knockout mice (Fig. 7A). H&E staining further confirmed more extensive inflammation in the skin of *Rev-erbα^-/-^* mice (Fig. 7B). Therefore, REV-ERBα has a regulatory role in the development of *P. acnes-*induced skin inflammation.

### REV-ERBα mediates regulation of *P. acnes*-induced inflammation by BMAL1

**Figure 8:**
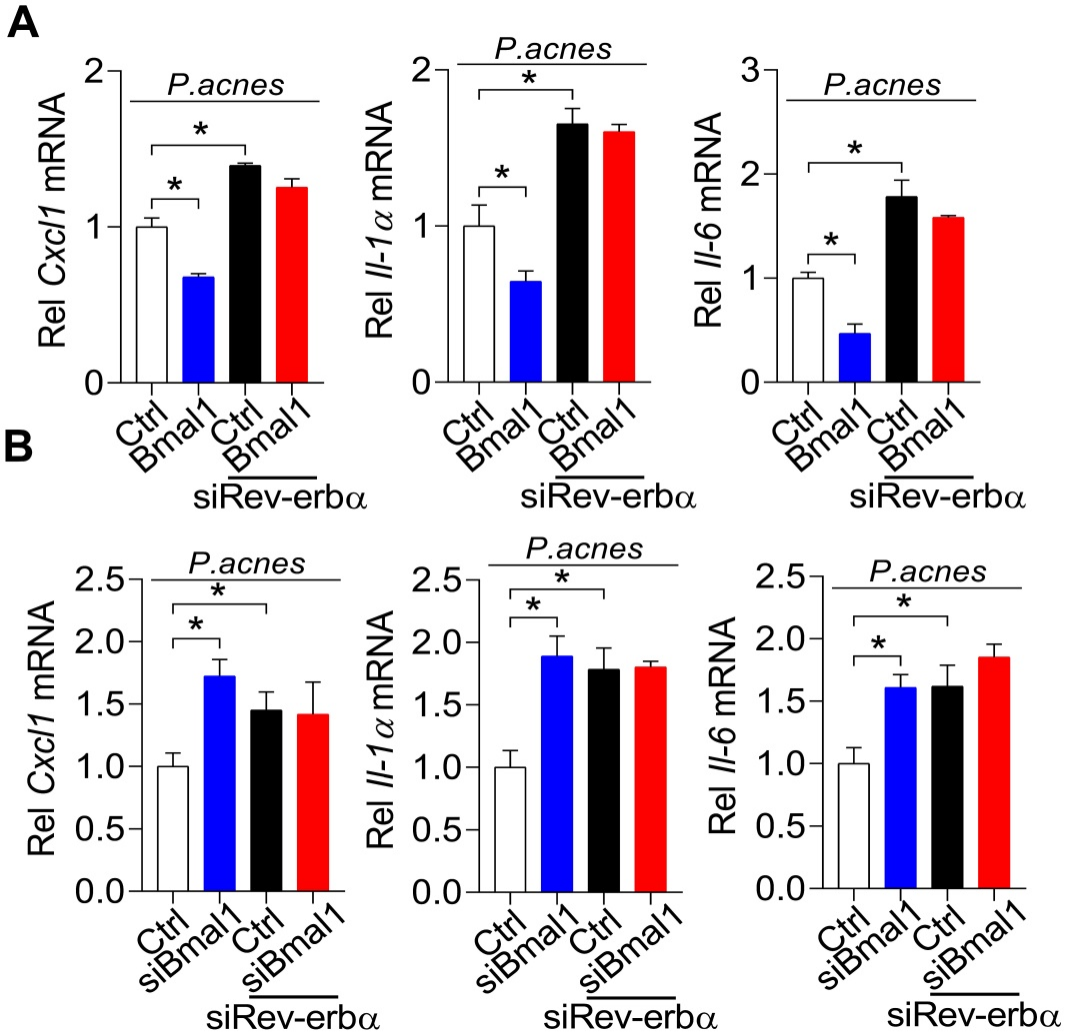
**REV-ERBα mediates regulation of *P. acnes*-induced inflammation by BMAL1. (A)** Effects of *Rev-erbα* knockdown on *Bmal1*-mediated inhibition of *Cxcl1*, *Il-1α* and *Il-6* mRNAs in *P. acnes*-treated primary mouse keratinocytes. Data are mean ± SD (*n* = 3). *p < 0.05 as determined by two-way ANOVA and Bonferroni post hoc test. **(B)** Effects of *Rev-erbα* knockdown on *Bmal1* knockdown-mediated induction of *Cxcl1*,* Il-1α* and *Il-6* mRNAs in *P. acnes*-treated primary mouse keratinocytes. Data are mean ± SD (*n* = 3). *p < 0.05 as determined by two-way ANOVA and Bonferroni post hoc test.

To further test whether REV-ERBα is involved in BMAL1 regulation of *P. acnes*-induced inflammation, we examined the influences of *Rev-erbα* silencing on regulatory effects of BMAL1 using primary mouse keratinocytes stimulated by *P. acnes*. As expected, *Rev-erbα* silencing led to increases in *Cxcl1*, *Il-1α* and *Il-6* (Fig. 8). Interestingly, *Rev-erbα* silencing attenuated the inhibitory effects of *Bmal1* overexpression on the expression of the pro-inflammatory factors* Cxcl1*, *Il-1α* and *Il-6* (Fig. 8A). Also, *Bmal1* knockdown failed to alter the expression of* Cxcl1*, *IL-1α* and *IL-6* in *Rev-erbα*-silenced cells (Fig. 8B). These findings support that BMAL1 regulates *P. acnes*-induced inflammation through REV-ERBα, a known target of BMAL1 and an inflammatory repressor.

## Discussion

In this study, we have observed marked disruption of clock gene expression in the skin of *P. acnes-*treated mice, particularly; the core clock gene *Bmal1* was down-regulated (Fig. 1). This suggested a role of *Bmal1* in acne development. Further, loss of *Bmal1* gene or chronic jet lag sensitized mice to *P. acnes*-induced skin inflammation. Chronic jet lag is regarded as a model of circadian dysfunction in which *Bmal1* is disrupted in most tissues and organs 27. Moreover, regulation of *P. acnes*-induced inflammation by *Bmal1* was confirmed in RAW264.7 cells and primary mouse keratinocytes. Mechanistically, *Bmal1* regulated *P. acnes*-induced skin inflammation via its target REV-ERBα, which acts on the NF-κB/NLRP3 axis to repress inflammation. Overall, our study provides a molecular mechanism for regulation of acne-associated inflammation by the skin clock, and suggests circadian rhythm as an intervention target for acne therapy.

In addition to *Bmal1*, expression of other clock genes such as *Rev-erbα, Dbp, Per1* and* Cry2* is also reduced in the skin of *P. acnes-*treated mice (Fig. 1). Expression change in *Bmal1* (down-regulation) should precede the changes in *Rev-erbα, Dbp, Per1* and* Cry2* (down-regulation) because *Bmal1* is a direct driver of these four clock genes 28. We thus propose that *Bmal1* is a critical component linking circadian clock to acne-associated inflammation. The finding that *Bmal1* is a repressor of acne-associated inflammation herein helps us to understand why circadian disruption is a contributor to acne development. However, why skin *Bmal1* is down-regulated in the acne model remains unaddressed. It is speculated that the acne-associated inflammation can in turn alter clock gene expression via the inflammatory cytokines such as TNF-α and IL-1β 29,30.

We have mainly investigated the role of *Bmal1* in skin inflammation in the acne model because the inflammation plays a central role in acne development, although other factors such as excess sebum production, follicular hyperkeratinization and formation of *Propionibacterium* colonies are also involved 31. This is supported by the fact that anti-inflammatory therapy remains a major component of the treatment of moderate to severe inflammatory acne 32. Farrah and Tan stated that acne is not an infectious disease, and reducing the burden of *P. acnes* does not equate to clearance of acne lesions 33. We also observed a positive correlation between the extent of skin inflammation and the acne severity (data not shown).

Based on transcriptomic analyses, we have demonstrated that NF-κB signaling and NLRP3 inflammasome pathway were involved in *P. acnes*-induced and *Bmal1*-regulated skin inflammation (Fig. 5). These findings are consistent with prior studies in which *P. acnes*-induced inflammation can be alleviated by inhibiting NF-κB signaling or NLRP3 inflammasome pathway 34. For example, tanshinone IIA, polyphyllin I and lactoferrin can ameliorate *P. acnes*-induced inflammation through suppressing NF-κB signaling 35-37. Licochalcone A and auranofin attenuate *P. acnes*-induced inflammation and acne symptoms through inactivation of NLRP3 inflammasome 38,39. REV-ERBα, a direct target of BMAL1, was previously shown to repress inflammation through the NF-κB/NLRP3 axis using a transrepression mechanism 19. Because the NF-κB/NLRP3 axis is also involved in BMAL1 regulation of *P. acnes*-induced skin inflammation, we thus have tested whether REV-ERBα has a mediating role in the regulation of skin inflammation by BMAL1. We have shown that 1) loss of *Rev-erbα* in mice exacerbates *P. acnes*-induced skin inflammation, a phenotype similar to that of *Bmal1^-/-^* mice, 2) *Rev-erbα* silencing attenuates the inhibitory effects of *Bmal1* overexpression on the expression of the pro-inflammatory factors* Cxcl1*, *IL-1α* and *IL-6*, and 3) *Bmal1* knockdown fails to alter the expression of* Cxcl1*, *IL-1α* and *IL-6* in *Rev-erbα*-silenced cells. Therefore, we propose that *Bmal1* restrains *P. acnes*-induced skin inflammation via its target REV-ERBα, which acts on the NF-κB/NLRP3 axis to repress inflammation. It is noteworthy that in a prior study with breast cancer cells, BMAL1 activates the NF-κB signaling pathway by promoting phosphorylation of IκB, recruitment of CBP (CREB binding protein) and acetylation of p65 40. Although the exact reason remains unknown as to why BMAL1 demonstrates opposing effects on NF-κB signaling pathway, there is a possibility that the type of BMAL1 action is tissue- and cell type-dependent. This is because the activity of BMAL1 is strongly affected by the cellular microenvironments such as the redox state and the types of cofactors 41.

To assess the effect of *Bmal1* on acne-associated inflammation, we used a mouse model of acne induced by *P. acnes*. This acne model is widely used and shows a similar pathological pattern (e.g., inflammatory response, epidermal thickening and formation of microcomedone-like cysts) to that of human acne 20. *P. acnes* is a gram-positive anaerobia that plays an essential role in initiating acne inflammation 42. Initiation of skin inflammation by *P. acnes* involves activation of inflammatory cells, monocytes, keratinocytes and sebocytes and subsequent secretion of inflammatory cytokines and chemokines such as IL-1α, IL-1β, IL-6, IL-8 and TNF-α 43-45. Of note, mouse CXCL1 (IL-8/CXCL8 in humans) is a member of CXC chemokine family and has a chemotactic activity for neutrophils, a major cell type in inflammatory skin lesions 45,46.

In summary, *Bmal1* disruption is identified as a potential pathological factor of acne-associated inflammation. Mechanistically, *Bmal1* regulates *P. acnes*-induced skin inflammation via its target REV-ERBα, which acts on the NF-κB/NLRP3 axis to repress inflammation. These findings increase our understanding of the crosstalk between skin clock and acne. Targeting circadian rhythms may represent a promising approach for management of acne.

## Figures and Tables

**Table 1:** Oligonucleotides used in this study

	Forward (5'-3' sequence)	Reverse (5'-3' sequence)
**Genotyping**		
*Bmal1^-/-^*	AGCGACTTCATGTCTCCG	GCTCTTACTAGTCAGTGGCA
*Rev-erbα^-/-^*	TCAGCTACAACTCCACACCG	CCCTGGCGTAGACCATTCAG
**RT-qPCR**		
*Cxcl1*	GACCATGGCTGGGATTCACC	CGCGACCATTCTTGAGTGTG
*Il-1α*	ACGTCAAGCAACGGGAAGAT	AAGGTGCTGATCTGGGTTGG
*Il-1β*	GGCTGTATTCCCCTCCATCG	AAGGTGCTGATCTGGGTTGG
*Il-6*	CCAGTGACTGAAAGACGCAT	AGATCCCTTACTCTCAGTCCAGAA
*Tnf-α*	CAGGCGGTGCCTATGTCTC	CGATCACCCCGAAGTTCAGTAG
*Bmal1*	CTCCAGGAGGCAAGAAGATTC	ATAGTCCAGTGGAAGGAATG
*Rev-erbα*	TTTTTCGCCGGAGCATCCAA	ATCTCGGCAAGCATCCGTTG
*Dbp*	ACATCTAGGGACACACCCAGTC	AAGTCTCATGGCCTGGAATG
*Per1*	GAAAGAAACCTCTGGCTGTTCC	GCTGACGACGGATCTTTCTTG
*Cry2*	GATGCCGATTTCAGTGTGAATG	GGCAGTAGCAGTGGAAGAAT
*Gapdh*	CAAGGAGTAAGAAACCCTGGACC	CGAGTTGGGATAGGGCCTCT
*β-actin*	GGCTGTATTCCCCTCCATCG	CCAGTTGGTAACAATGCCATGT
